# Affective, cognitive, and arousal responses to Marko Kraljević and Alija Đerzelez: a cross-cultural study of narrative perception among multicultural students

**DOI:** 10.3389/fpsyg.2026.1798180

**Published:** 2026-05-25

**Authors:** Kristina Mitić, Emina Saračević, Petar Mitić, Nikola Stojanović, Nebojša Trajković

**Affiliations:** 1 Faculty of Philosophy, Department of Serbian Language and Literature, University of Niš, Niš, Serbia; 2 Faculty of Sport and Physical Education, University of Niš, Niš, Serbia

**Keywords:** collective memory, epic poetry, intergroup evaluation, narrative engagement, social identity theory

## Abstract

**Background:**

Epic poetry represents an important component of literary education and plays a significant role in shaping historical consciousness and cultural identity among students in multicultural learning environments. The aim of this study was to examine students' affective, cognitive, and arousal responses to two selected epic narratives centered on Marko Kraljević and Alija Đerzelez within an educational context, and to explore how students' cultural background moderated responses to these specific narrative stimuli.

**Methods:**

Using a mixed factorial design, 64 seventh-grade students (32 Serbian, 32 Bosniak) from multicultural schools evaluated both poems. Responses were analyzed using linear mixed-effects models with poem, ethnicity, and their interaction as fixed effects and participant as a random intercept.

**Results:**

Significant poem × ethnicity interactions were observed for valence, *F*_(1, 62)_ = 42.17, *p* < 0.001; cognitive evaluation, *F*_(1, 62)_ = 53.01, *p* < 0.001; and arousal, *F*_(1, 62)_ = 40.50,*p* < 0.001. Simple effects showed that Bosniak students evaluated Alija more positively in valence and cognitive terms, whereas Serbian students favored Marko. Arousal differed only among Serbian students, who reported stronger engagement with Marko, while Bosniak students showed no arousal difference.

**Conclusions:**

Although both selected narratives were generally evaluated positively, students' responses differed in the relative strength of affective, cognitive, and arousal evaluations as a function of cultural background. These findings underscore the importance of critically contextualizing national literary traditions in educational settings to promote reflective and inclusive engagement with shared cultural heritage.

## 1 Introduction

Social identity develops through direct social interaction, but it is also shaped by symbolic systems embedded in language, culture, and shared stories. Beyond face-to-face interaction, individuals encounter collective meanings through culturally transmitted narratives that provide interpretative frameworks for understanding social belonging. Culturally salient forms of storytelling, such as epic poetry, can serve as psychological stimuli that influence how individuals feel, interpret meaning, and orient toward in-group and out-group symbols. Narratives operate not only as carriers of information but also as affective and evaluative structures that organize emotional responses and moral positioning toward social groups (Green and Brock, 2000; Mar et al., 2011; Bouizegarene et al., 2024). The importance of such symbolic material for identity formation has long been emphasized in social identity and nationalism research (Smith, 2004, 2009; Tajfel and Turner, 2004). These processes are particularly relevant in educational contexts, where students encounter culturally charged material during ongoing phases of identity development and where narratives embedded in curricula may function as implicit cues for social identification (Hammack, 2010).

Language plays a central role in shaping how culturally salient content is interpreted, as meaning-making is embedded in broader linguistic and sociocultural frameworks rather than residing solely in lexical comprehension (Busch, 2012; Hammack, 2010). Serbian and Bosniak belong to the same South Slavic linguistic continuum and show a high degree of mutual intelligibility (Greenberg, 2008). However, shared linguistic structure primarily supports surface-level comprehension and does not necessarily imply convergence in interpretation or evaluative response when texts are symbolically and historically charged. Sociolinguistic and discourse-oriented research indicates that shared linguistic forms do not guarantee shared meaning, particularly when language is embedded in narratives linked to collective memory and group identity (Busch, 2012; Greenberg, 2008). Meaning thus emerges from culturally situated interpretative frames that guide emotional appraisal and evaluative judgment (Busch, 2012; Hammack, 2010). These dynamics are particularly salient in multiethnic educational settings, where students share classroom environments while drawing on partially distinct cultural and historical reference frameworks. Research on education in divided or post-conflict societies shows that students' engagement with literary and historical texts is shaped more strongly by interpretative frameworks linked to identity and collective memory than by differences in language proficiency (Barton, 2001; Barton and McCully, 2007). Recent systematic analyses of ethnically differentiated curricula in post-war Bosnia and Herzegovina further demonstrate how educational materials contribute to the construction and maintenance of “us” and “them” boundaries within shared institutional settings (Kovač, 2025), underscoring that symbolic differentiation may persist even when schooling structures are formally integrated. In such contexts, institutional arrangements that support minority language education coexist with shared curricula, creating situations in which the same texts may be linguistically accessible yet symbolically contested (Barton and McCully, 2007). Empirical studies further indicate that tensions surrounding literature and history instruction often arise from divergent cultural interpretations rather than from limited comprehension per se (Barton, 2001; Cernak and Beljanski, 2022).

Within this context, epic poetry occupies a central place in both Serbian and Bosniak literary traditions. From a behavioral science perspective, epic poems can be understood as culturally transmitted narratives that convey group-relevant meanings, moral evaluations, and symbolic representations of social identity (Hammack, 2010; Smith, 2004). Research in cultural and narrative psychology indicates that such narratives function as repositories of collective memory and provide interpretative frameworks through which individuals evaluate social symbols and group membership (Hammack, 2010; Wertsch, 2008). Through recurring narrative structures and stable heroic figures, epic poetry contributes to the intergenerational transmission of values and conceptions of belonging. Engagement with these texts is not uniform or passive, as readers actively interpret heroic figures in relation to their own social identity and prior cultural knowledge, a process documented in research on narrative identity and meaning-making (Hammack, 2010; McLean and Syed, 2015). Accordingly, epic poetry may be approached as a psychologically active stimulus capable of eliciting affective responses, evaluative judgments, and varying levels of engagement. Empirical research further shows that identity-relevant narratives influence affective engagement and evaluative processing, particularly in intergroup contexts where stories are tied to moral positioning and historical meaning (Green and Brock, 2000; Hammack, 2010). Narrative engagement has been shown to intensify emotional involvement when stories resonate with the reader's social identity (Green and Brock, 2000; Mar et al., 2011), and recent empirical research further demonstrates that transportation and identity alignment interact to shape affective involvement in narrative contexts (Wang et al., 2024).

Although Serbian and Bosniak epic poetry has been extensively examined from literary, historical, and philological perspectives, existing scholarship has predominantly focused on textual structure, symbolism, and historical context, with comparatively limited attention to how these texts are psychologically experienced by contemporary students in shared educational environments (Smith, 2004; Wertsch, 2008). Research in education and social psychology has focused primarily on students' interpretations, attitudes, and explicit judgments about culturally salient texts, particularly in relation to history and national narratives (Barton, 2001; Barton and McCully, 2007). Far fewer studies have examined how students from different ethnic backgrounds emotionally engage with and cognitively evaluate literary narratives that are simultaneously familiar, symbolically loaded, and identity-relevant (Hammack, 2010; Mar et al., 2011). This gap is important because epic poetry continues to occupy a visible place in educational curricula and informal cultural transmission. At the same time, its role as a psychologically active stimulus in intergroup contexts remains insufficiently examined. Research on social identity and narrative engagement suggests that culturally salient texts may elicit group-specific patterns of affective response and evaluative processing even when linguistic comprehension is shared (Green and Brock, 2000; Hammack, 2010; Tajfel and Turner, 2004). However, much of this literature has emphasized explicit evaluations, with comparatively less attention to affective responses and engagement intensity, such as emotional valence and arousal, elicited by literary narratives in everyday school settings (Green and Brock, 2000; Mar et al., 2011).

Therefore, the present study aimed to examine students' affective, cognitive, and arousal responses to two selected epic narratives centered on Marko Kraljević and Alija Đerzelez within an educational context. Rather than treating these responses as representative of epic heroes or national epic traditions in general, the study focuses on how students from different cultural backgrounds responded to these two specific narrative stimuli. Rather than approaching epic poetry as a neutral curricular artifact, the study conceptualizes it as a psychologically active cultural medium whose reception may differ across groups even within shared educational environments (Green and Brock, 2000; Hammack, 2010; Tajfel and Turner, 2004). Based on this framework, two hypotheses were formulated. First, students were expected to evaluate the selected poem associated with their own cultural tradition more positively, in terms of valence and cognitive evaluation, than the selected poem associated with the other group. Second, the selected poem aligned with students' in-group tradition was hypothesized to elicit higher levels of arousal, reflecting stronger engagement with identity-relevant narrative material (Green and Brock, 2000; Mar et al., 2011; Russell, 1980; Barrett and Russell, 2014).

## 2 Materials and methods

### 2.1 Participants

The study included 64 seventh-grade students from the Raška District in south-western Serbia (32 Bosniak and 32 Serbian). The sample comprised 33 girls and 31 boys, with comparable gender distribution across ethnic groups (Bosniak: 15 girls, 17 boys; Serbian: 18 girls, 14 boys). The sample was balanced for ethnic background, ensuring comparability across groups. Participants were recruited from multicultural schools in which Bosniak and Serbian students attend classes together, reflecting the everyday educational context of the region. The Raška District is characterized by a heterogeneous ethnic composition, with Bosniaks and Serbs representing the predominant groups. Although Serbian is the official language of instruction, the education system in Serbia provides institutional support for instruction in minority languages in municipalities with substantial minority populations, including Bosniak-language education where demand exists (European Agency for Special Needs and Inclusive Education, 2023). In this context, Bosniak-language instruction has been established in several municipalities within the Raška District as part of the regular public education system. This sociocultural setting is marked by long-standing coexistence between Bosniak and Serbian communities. While the broader historical context of the conflicts in the former Yugoslavia has influenced interethnic relations in the region, everyday social and educational interactions in the Raška District are characterized by regular contact and shared institutional environments. The present sample, therefore, reflects a context in which students are routinely exposed to both cultural traditions. Prior to participation, written informed consent was obtained from parents or legal guardians. Students' ethnicity was self-reported and confirmed during the consent procedure. Ethnicity was operationalized as self-identified group membership; the strength or centrality of ethnic identity was not assessed. Ethical approval for the study was granted by the Ethics Committee of the Faculty of Philosophy, University of Niš (Decision No. 17-2024, approved on 14 December 2024). All procedures were conducted in accordance with the ethical standards of the institutional research committee and the principles of the Declaration of Helsinki. An a priori power analysis was conducted to determine the required sample size to detect interaction effects in a mixed-design model. The obtained sample met the estimated requirements for adequate statistical power.

### 2.2 Stimuli

Two epic folk poems were selected, one representing the Bosniak tradition (Đerzelez Alija, carev mejdandžija; Marjanović, 1898) and one representing the Serbian tradition (Marko Kraljević and Musa Kesedžija; &##1055;&##1077;&##1096;&##1080;&##1115;, 1988). From each poem, a thematically comparable excerpt was selected, depicting the central heroic figure confronting and defeating an opponent of a different religious background. It should be noted that each heroic figure was represented by a single selected poem excerpt. Thus, the stimuli were designed to provide a controlled comparison between two specific narrative representations rather than to capture the full range of depictions of either heroic figure across their respective epic traditions. The selected excerpts were comparable in length, narrative structure, and thematic focus, and were chosen to preserve a parallel depiction of conflict, resolution, and heroic agency across traditions. This choice was intended to reduce differences in aesthetic and thematic organization while keeping the focus on the central heroic figures. The texts were presented in their standard literary form as commonly used in educational materials, without substantive modification. Both poems are widely recognized within their respective literary traditions and are commonly encountered in school curricula and broader cultural transmission. Marko Kraljević and Alija Đerzelez were selected because they are among the most recognizable heroic figures within their respective epic traditions and are associated with substantial bodies of epic material. It should be noted, however, that each heroic figure was represented by a single selected poem excerpt. Thus, the stimuli were designed to provide a controlled comparison between two specific narrative representations rather than to capture the full range of depictions of either heroic figure across their respective epic traditions.

### 2.3 Procedure

Poems were presented in both written and oral form during regular school hours in a classroom setting. Participants first listened to the audio presentation of each poem excerpt individually and were simultaneously provided with the written text to ensure comprehension. After exposure to each poem, students completed the questionnaire assessing their subjective responses before proceeding to the next poem. The order of poem presentation was randomized across participants to minimize potential order and carryover effects. Each participant evaluated both poems within the same session, allowing for direct within-subject comparison while maintaining independence of questionnaire responses for each stimulus.

### 2.4 Research design

The study employed a mixed factorial design with poem origin (Bosniak vs. Serbian) as a within-subject factor and participant ethnicity (Bosniak vs. Serbian) as a between-subjects factor. This design enabled examination of (a) main effects of poem origin, (b) main effects of participant ethnicity, and (c) the interaction between poem origin and ethnicity. The primary analytical focus was the interaction term, which tested whether the relative evaluation of the two poems differed as a function of participants' ethnic background.

### 2.5 Measures

Participants' subjective responses were assessed using a questionnaire based on the connotative differential method (Janković, 2000), which captures experiential meaning through bipolar semantic scales. Each poem was rated using 15 semantic differential items (e.g., unpleasant–pleasant, meaningless–meaningful), grouped into three theoretically derived dimensions: emotional evaluation, cognitive evaluation, and arousal. Emotional evaluation reflected affective responses to the poem, including perceived pleasantness, attractiveness, beauty, and goodness (Cronbach's α = 0.87–0.90 across poems, average inter-item *r* = 0.58–0.66). Cognitive evaluation assessed perceived meaningfulness, comprehensibility, familiarity, logical coherence, and clarity (Cronbach's α = 0.87, average inter-item *r* = 0.58). Arousal captured the degree of engagement and stimulation elicited by the poem, including ratings of remarkableness, stimulation, interest, motivation, and perceived significance (Cronbach's α = 0.86, average inter-item *r* = 0.56). Consistent with dimensional models of affect, arousal is treated here as the activation component of subjective experience rather than a separate emotion category (Russell, 1980; Barrett and Russell, 2014). Responses were initially recorded on a −3 to +3 scale and subsequently rescaled to a 1–7 metric for statistical analysis, with −3 corresponding to 1 and +3 to 7. For each participant and poem, dimension scores were computed as the mean of the items corresponding to that dimension.

### 2.6 Statistical analysis

Descriptive statistics (means and standard deviations) were calculated for all dimension scores separately for each poem and ethnic group. Inferential analyses were conducted using linear mixed-effects models to account for the repeated-measures structure of the data. Separate models were fitted for each evaluative dimension (emotional evaluation, cognitive evaluation, and arousal), with poem origin (Bosniak vs. Serbian) specified as a within-subject factor, participant ethnicity (Bosniak vs. Serbian) as a between-subject factor, and their interaction included as a fixed effect. Participant identity was entered as a random intercept to model within-subject dependence arising from evaluations of both poems. Models were estimated using restricted maximum likelihood. The primary focus of inference was the poem × ethnicity interaction, which tested whether the relative evaluation of the two poems differed as a function of participants' ethnic background. When significant interactions were present, simple effects were examined using estimated marginal means, comparing poems within each ethnic group and ethnic groups within each poem. *p*-Values for simple effects were adjusted for multiple comparisons using the Benjamini–Hochberg procedure. Effect sizes for simple effects were expressed as standardized mean differences (Cohen's *d* ), calculated as the estimated contrast divided by the residual standard deviation (σ) of the corresponding mixed-effects model, with 95% confidence intervals derived from the associated standard errors and degrees of freedom. Effect sizes were interpreted using conventional benchmarks: values of approximately 0.2, 0.5, and 0.8 indicate small, moderate, and large effects, respectively (Cohen, 1988). Model assumptions were evaluated by inspecting residual plots for linearity and homoscedasticity, and by quantile–quantile plots of residuals to assess departures from normality. No substantial violations were observed that would compromise model validity, and influence diagnostics did not indicate undue leverage of individual observations. All analyses were conducted in R (version 4.2.0; RStudio version 2025.09.2 + 418; RStudio, Posit Software, PBC, Boston, MA, United States), using the lme4 package (Bates et al., 2015).

## 3 Results

Table 1 presents descriptive statistics for all measured variables across ethnicity and poem. Mean scores and standard deviations are reported for items within the Valence, Cognitive, and Arousal dimensions, separately for Bosniak and Serbian participants and for both poems (Bosniak and Serbian). These descriptive results provide an overview of central tendency and variability across experimental conditions and participant groups. Inferential analyses were subsequently conducted using linear mixed-effects models to account for the repeated-measures structure of the data and to examine differences associated with poem, nationality, and their interaction.

**Table 1:** Descriptive statistics for valence, cognitive evaluation, and arousal across participant ethnicity and poem.

Dimension	Bosniak		Serbian	
	Bosniak poem	Serbian poem	Bosniak poem	Serbian poem
Valence				
Unpleasant/pleasant	5.34 ± 1.27	4.91 ± 1.59	4.5 ± 1.23	5.84 ± 0.94
Repulsive/attractive	5.12 ± 1.22	4.44 ± 1.56	3.84 ± 1.04	5.72 ± 1.16
Disliked/liked	5 ± 1.4	4.22 ± 1.64	4.38 ± 1.2	5.69 ± 1.13
Ugly/beautiful	5.69 ± 1.24	4.84 ± 1.74	4.81 ± 1.21	5.56 ± 1.2
Bad/good	5.38 ± 1.2	4.78 ± 1.39	4.94 ± 1.17	5.62 ± 1.14
Dimension mean score	5.31 ± 0.91	4.64 ± 1.39	4.49 ± 0.9	5.69 ± 0.86
Cognitive				
Meaningless/meaningful	5.66 ± 1.29	4.78 ± 1.71	4.41 ± 2.08	5.66 ± 1.29
Incomprehensible/comprehensible	4.72 ± 1.91	4.84 ± 1.81	2.59 ± 2.03	5.91 ± 1.07
Illogical/logical	5.06 ± 1.58	4.31 ± 1.82	3.84 ± 1.99	5.41 ± 1.25
Unfamiliar/familiar	5.09 ± 1.74	3.94 ± 1.81	3.38 ± 2.17	6.03 ± 1.29
Unclear/clear	5.28 ± 1.31	3.94 ± 1.86	4.22 ± 1.77	5.62 ± 1.25
Dimension mean score	5.16 ± 1.16	4.36 ± 1.27	3.69 ± 1.65	5.72 ± 0.82
Arousal				
Unremarkable/remarkable	5 ± 1.52	4.56 ± 1.81	4.56 ± 1.17	5.62 ± 0.99
Unstimulating/stimulating	4.53 ± 1.57	4.09 ± 1.47	4.31 ± 1.51	4.97 ± 1.21
Boring/interesting	5 ± 2.02	4.5 ± 1.88	3.66 ± 1.71	5.53 ± 1.09
Unmotivating/motivating	4.12 ± 1.82	3.88 ± 1.62	3.75 ± 1.86	5.12 ± 1.3
Insignificant/significant	4.56 ± 2.38	4.59 ± 2	2.78 ± 1.97	5.91 ± 1.67
Dimension mean score	4.64 ± 1.3	4.32 ± 1.41	3.81 ± 1.09	5.43 ± 0.97

Values represent mean ± SD for each item, separately reported for Bosniak and Serbian participants evaluating Bosniak and Serbian poems. Dimension mean scores represent the mean score of all items belonging to the corresponding dimension.

Before considering group-specific differences, it is important to note that both selected epic narratives were generally evaluated positively by both groups. Across most valence and cognitive evaluation items, mean scores were above the midpoint of the scale, indicating that both Marko Kraljević and Alija Đerzelez were generally perceived as positive, meaningful, and recognizable heroic figures. Thus, the observed interaction effects should be interpreted as relative differences in the strength of positive evaluation rather than as evidence that one group rejected or negatively evaluated the other group's narrative figure.

**Figure 1:**
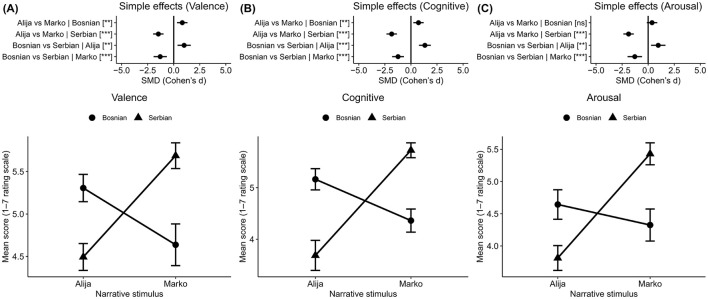
Combined visualization of simple effects and interaction patterns for **(A)** valence, **(B)** cognitive evaluation, and **(C)** arousal. For each panel, the upper plot displays standardized mean differences (SMD; Cohen's d) with confidence intervals, illustrating contrasts between poems within each ethnic group and between ethnic groups within each poem. The lower plot shows mean scores on the 1–7 rating scale with standard errors for each narrative stimulus, separately for Bosnian and Serbian participants, highlighting the interaction patterns between poem origin and participant ethnicity. Together, the panels allow visual comparison of the direction, magnitude, and consistency of group-specific evaluative responses across the three dimensions. Shapes indicate nationality, with circles (•) representing Bosnian participants and triangles (▴) representing Serbian participants. Error bars show standard errors (SE). Significance markers (**, ***) denote significant differences between songs within each nationality (**p < 0.01, ***p < 0.001).

Because each heroic figure was represented by a single selected poem excerpt, the results are reported as responses to the two specific narrative stimuli rather than as generalized responses to Serbian or Bosniak epic poetry as a whole. Linear mixed-effects models revealed statistically significant interactions between poem and nationality for all three evaluative dimensions: valence, *F*_(1, 62)_ = 42.17, *p* < 0.001; cognitive evaluation, *F*_(1, 62)_ = 53.01, *p* < 0.001; and arousal, *F*_(1, 62)_ = 40.50,*p* < 0.001. These interactions indicate that, within an overall pattern of generally positive evaluation, the relative appraisal of the two selected narratives differed as a function of participants' ethnic background. Given the presence of interaction effects, interpretation focused exclusively on conditional (simple) effects rather than marginal main effects (see Figure 1).

For valence, Bosniak participants evaluated the Bosniak poem more positively than the Serbian poem, *p* = 0.002, with a large standardized effect size (*d* = 0.82). In contrast, Serbian participants exhibited the opposite pattern, rating the Serbian poem more positively than the Bosniak (*p* < 0.001), with a large effect size (*d* = −1.47). Between-group comparisons further indicated that Bosniak participants rated the Bosniak poem more positively than Serbian participants (*d* = 1.00; large effect). In contrast, Serbian participants rated the Serbian poem more positively than Bosniak participants (*d* = −1.29, large effect).

A comparable crossover pattern was observed for cognitive evaluation. Bosniak participants rated the Bosniak poem as more meaningful and comprehensible than the Serbian, *p* = 0.005, corresponding to a moderate effect size (*d* = 0.73). Serbian participants again showed the reverse pattern, rating the Serbian poem substantially higher than the Bosniak poem (*p* < 0.001), with a large effect size (*d* = −1.85). Comparisons between nationalities revealed that Bosniak participants rated the Bosniak poem higher than Serbian participants (*d* = 1.34, large effect), whereas Serbian participants rated the Serbian poem higher than Bosniak participants (*d* = −1.24, large effect).

Finally, the arousal simple effects reflected divergent evaluative patterns across nationalities. Among Bosniak participants, the difference in arousal between the two poems was not statistically significant (*p* = 0.14), and the effect size was small (*d* = 0.37). In contrast, Serbian participants found the Serbian poem substantially more arousing than the Bosniak,*p* < 0.001, with a large effect size (*d* = −1.88). Between-group comparisons showed that Bosniak participants reported higher arousal to the Bosniak poem than Serbian participants (*d* = 0.97, large effect). In contrast, Serbian participants reported higher arousal to the Serbian poem (*d* = −1.28, large effect).

## 4 Discussion

This study examined how Serbian and Bosniak students responded affectively, cognitively, and experientially to two selected epic narratives centered on Marko Kraljević and Alija Đerzelez. The first point to emphasize is that both selected heroic figures were generally evaluated positively by both groups of students. Across the descriptive results, mean scores for both narratives were mostly above the midpoint of the scale, particularly for valence and cognitive evaluation, indicating that neither figure was experienced primarily in negative or rejecting terms. Thus, the observed effects should be interpreted as relative differences in the strength of positive evaluation rather than as evidence of rejection of the other group's heroic figure. Within this broader pattern of generally positive appraisal, the significant poem × ethnicity interactions suggest that students' evaluations varied according to the alignment between their ethnic background and the cultural origin of the selected narrative. This pattern is consistent with social identity theory, which posits that individuals tend to evaluate in-group symbols more favorably to sustain a positive social identity (Tajfel and Turner, 2004), but it should be understood as relative in-group enhancement within an overall positive evaluative context rather than simple out-group devaluation. Within the broader cultural history of the Balkans, epic poetry can function as a symbolic repository of collective memory and identity, contributing to the formation and maintenance of ethnic affiliation and historical consciousness (Koljević, 1980; Detelić et al., 2015; Smith, 2004; Wertsch, 2008). From this perspective, the two selected narratives may be understood as culturally situated stimuli whose reception was shaped by both shared literary recognition and identity-relevant interpretative frames (Hogg, 2016). We should note that the within-subject presentation of both poems may have encouraged comparative processing, and this design feature could have amplified perceived differences between narratives through contrast effects. However, contrast-based amplification would be expected to operate similarly across participants. The fact that differentiation depended on the alignment between the poem's cultural origin and the participant's ethnicity suggests that the pattern is not readily attributable to a generic contrast mechanism alone, although the magnitude of the effects should be interpreted with this design feature in mind.

This finding aligns with broader work on national imagination and symbolic affiliation, which emphasizes that shared cultural narratives contribute to the construction of perceived group boundaries and collective belonging (Anderson, 2020; Hammack, 2010; Smith, 2009). Notably, the present results extend this literature by demonstrating that such processes are evident not only in explicit attitudes or historical interpretations but also in immediate affective and cognitive responses elicited by literary material in an educational context. From a behavioral science perspective, these group-specific patterns can be interpreted as differences in appraisal and activation during narrative processing, driven by identity-linked schemas that shape how culturally familiar and unfamiliar stimuli are evaluated. Epic poems serve here as symbolic stimuli that cue associative networks related to in-group and out-group representations, thereby influencing affective appraisal (valence), perceived meaning and coherence (cognitive evaluation), and subjective activation (arousal) (Hammack, 2010; Tajfel and Turner, 2004). In this framing, collective memory is not treated as an abstract cultural force but as psychologically instantiated expectations and interpretative frames acquired through social learning, which bias attention and evaluation toward identity-relevant cues (Hammack, 2010; Wertsch, 2008). Narrative engagement models further suggest that when a story resonates with the reader's identity-relevant knowledge structures, it becomes more likely to elicit stronger evaluative and emotional responses, even when the text is equally comprehensible at the linguistic level (Green and Brock, 2000; Mar et al., 2011). This interpretation is consistent with social identity accounts, which emphasize that in-group symbolic material tends to receive preferential evaluation because it supports positive social identification (Hogg, 2016; Tajfel and Turner, 2004).

In contrast to the convergent patterns observed for affective and cognitive evaluations, arousal responses, conceptualized here as activation, displayed a marked asymmetry across groups. Whereas Serbian students reported higher engagement with the epic narrative aligned with their own cultural tradition, Bosniak students did not exhibit a comparable differentiation in arousal across poems. This dissociation between evaluative judgment and arousal suggests that positive appraisal of a culturally familiar narrative does not necessarily translate into heightened experiential activation (Scherer, 2005). From the perspective of narrative psychology, arousal may be understood as reflecting the degree to which a narrative elicits immersive engagement rather than evaluative endorsement alone (Green and Brock, 2000; Mar et al., 2011). Prior work on narrative transportation indicates that stories emphasizing struggle, tension, and action are more likely to elicit relatively heightened emotional activation within identity-relevant contexts, particularly when they resonate with the reader's identity-relevant schemas (Green and Brock, 2000; Oatley, 2016). Within this framework, the observed asymmetry suggests that engagement with epic poetry may depend not only on symbolic identification with a hero but also on the narrative modes through which that hero is culturally represented. Importantly, this interpretation does not imply a uniform hierarchy of emotional intensity across traditions. Rather, it highlights that distinct epic narratives may emphasize different dimensions of meaning, such as moral order, continuity, or conflict, which may differentially shape experiential engagement. Similar distinctions have been noted in research on collective memory, where narratives centered on endurance and moral exemplarity tend to support identity affirmation without necessarily eliciting heightened emotional arousal (Hammack, 2010; Wertsch, 2008). As such, variation in arousal responses may reflect differences in narrative emphasis rather than differences in the perceived value or significance of the cultural figures themselves.

One plausible explanation for the observed pattern of arousal among Serbian students lies in the cultural positioning of Marko Kraljević within Serbian and broader South Slavic epic traditions. Marko Kraljević occupies a uniquely central role in Serbian epic poetry, where he is repeatedly depicted as a figure of physical strength, moral ambivalence, and active struggle. Through extensive oral transmission and literary consolidation, this figure has become a dominant symbolic representation of resistance, endurance, and confrontation with historical adversity (Detelić et al., 2015; Koljević, 1980; Sang Hun and Hyok Jae, 2016). From the perspective of collective memory, such figures tend to function as emotionally activating symbols, condensing narratives of struggle, conflict, and agency into a recognizable heroic form (Halbwachs, 1992; Wertsch, 2008). Research on cultural memory suggests that narratives emphasizing action and confrontation are more likely to elicit heightened emotional engagement (Zillmann, 2006), particularly when they align with socially shared representations of historical experience (Hammack, 2010). In this sense, the stronger arousal responses observed among Serbian students may reflect the way in which the selected Marko Kraljević excerpt foregrounded themes of active resistance, confrontation, and moral tension, while also resonating with broader cultural representations of Marko Kraljević within Serbian epic tradition. We should note that this interpretation does not imply a direct causal link between specific narrative content and emotional response, nor does it suggest that arousal is an inherent property of the character itself. Rather, it points to the possibility that culturally reinforced narrative patterns surrounding certain heroic figures may shape how young readers engage with epic poetry at the experiential level, particularly when such narratives resonate with identity-relevant historical schemas.

By contrast, Alija Đerzelez occupies a different narrative position within Bosniak epic traditions. While he is widely recognized as a prominent heroic figure, representations of D*erzelez tend to emphasize moral authority, generosity, and communal order rather than sustained conflict or existential struggle. Within cultural memory frameworks, such figures often function as stabilizing symbols that reinforce continuity, ethical norms, and collective cohesion (Hammack, 2010; Wertsch, 2008). From the standpoint of narrative engagement, this mode of representation may support positive affective and cognitive evaluation without necessarily eliciting heightened arousal. Research in narrative psychology suggests that stories emphasizing moral exemplarity and social harmony can promote endorsement and identification (Aquino and Reed (2002)), whereas narratives structured around tension and confrontation may be more likely to elicit heightened emotional activation (Green and Brock, 2000; Oatley, 2016). In this context, the absence of differentiated arousal responses among Bosniak students may reflect the narrative functions associated with D*erzelez rather than diminished emotional significance. Importantly, this interpretation does not imply that Bosniak epic traditions are less emotionally resonant, nor that arousal constitutes a superior or more authentic form of engagement. Rather, it highlights that epic narratives may organize emotional experience along different dimensions, with some traditions foregrounding evaluative affirmation and moral meaning, and others emphasizing activation and struggle. Such variation is consistent with broader findings in collective memory research, which show that cultural narratives can sustain identity through multiple experiential pathways (Halbwachs, 1992; Wertsch, 2008).

Taken together, the present findings suggest that, within the specific sociocultural context examined, engagement with epic poetry in educational settings extends beyond literary comprehension and involves identity-relevant affective and experiential processes. Even when encountered outside ritualized or performative contexts, epic narratives appear capable of eliciting differentiated responses that align with students' social identities. This observation is consistent with research suggesting that culturally embedded texts can function as implicit carriers of collective meaning, shaping emotional engagement without explicit evaluative framing (Hammack, 2010; Wertsch, 2008). We argue that the emergence of group-specific patterns of evaluation and arousal in a shared classroom context indicates that exposure to standard curricular material does not necessarily entail uniform experiential processing. Prior work in educational psychology and narrative studies has shown that students may engage with the same texts through distinct interpretative frames grounded in cultural memory and social identification, particularly when such texts are embedded in school curricula that transmit historical and national narratives (Barton and McCully, 2007; Carretero et al., 2012; Hammack, 2010). The present findings extend this literature by demonstrating that such differentiation is evident not only in explicit interpretation or judgment, but also in affective and engagement-related dimensions that operate at a more immediate experiential level. Rather than implying a need for prescriptive curricular responses, these results highlight the analytical value of attending to how shared literary materials are experienced within pluralistic educational environments. Understanding that culturally salient narratives may activate multiple forms of engagement provides a more nuanced perspective on how identity and emotion intersect in everyday educational encounters, particularly in contexts characterized by overlapping but distinct historical narratives.

While the present study provides a controlled examination of affective, cognitive, and arousal responses to epic poetry, several limitations should be acknowledged. The sample was drawn from a single geographic region where epic narratives remain culturally salient, which may limit the generalizability of the findings to regions with different historical dynamics or identity configurations. Another important limitation concerns the restricted stimulus set used in the present study. Participants evaluated only two specific heroic figures, Marko Kraljević and Alija Đerzelez, with one figure selected from each literary tradition. These figures were selected because they are among the most recognizable heroes in their respective epic traditions and are associated with substantial bodies of epic material. Nevertheless, the inclusion of only one heroic figure from each tradition limits the extent to which the findings can be generalized to epic heroes as a broader category or to other heroic figures within Serbian and Bosniak epic traditions. In addition, each figure was represented by only one selected poem excerpt. This choice was made to preserve comparable plot structure and literary form across the two stimuli, thereby reducing aesthetic and thematic differences that could otherwise influence students' evaluations. However, this design also limits generalization to other poems featuring the same heroic figures, since Marko Kraljević and Alija Đerzelez may be represented differently across the wider epic corpus. The observed patterns should therefore be interpreted as responses to these two selected figures as represented in the specific narrative materials used in the study. Future research should include multiple heroic figures from each tradition and multiple poems or excerpts for each figure, to distinguish responses to individual heroes from responses to particular textual representations and from broader responses to culturally embedded heroic traditions. Nevertheless, the balanced representation of Bosniak and Serbian participants and the within-subject exposure to both poems support internal validity and allow for a sensitive assessment of interaction effects between poem origin and participant ethnicity.

A further limitation concerns the reliance on self-reported measures, which capture subjective experience but do not directly index physiological or automatic emotional responses. Moreover, ethnicity was operationalized as self-identified group membership rather than as a multidimensional measure of ethnic identity centrality or commitment. Future research may incorporate validated ethnic identity scales and multi-method approaches to examine how identity strength moderates narrative engagement. Although such measures are well-suited to assessing experiential meaning, future research may benefit from integrating psychophysiological indicators, such as heart rate variability or skin conductance, to provide convergent evidence of emotional engagement. In addition, the focus on epic poetry as a specific literary form necessarily constrains the scope of inference. The study compared two culturally salient epic narratives without including a neutral baseline condition; thus, the findings speak to relative differences in engagement across identity-aligned and non-aligned narratives rather than to absolute levels of activation relative to neutral literary material. Future studies could extend the present framework by incorporating neutral or intra-group narratives, as well as other culturally salient forms such as historical texts or audiovisual media, to provide a broader contextual benchmark and to examine whether similar patterns of identity-related engagement emerge across modalities. Despite these limitations, the present study demonstrates that epic poetry appears to function as a psychologically active medium in contemporary educational contexts, capable of eliciting differentiated evaluative and engagement-related responses aligned with social identity. By combining a mixed-design approach with fine-grained assessment of experiential dimensions, the study contributes to a more nuanced understanding of how cultural narratives shape subjective experience in pluralistic settings.

## 5 Conclusions

The present study showed that both selected epic narrative excerpts were generally evaluated positively by Serbian and Bosniak students, indicating that the two heroic figures were not experienced primarily in negative or rejecting terms. Against this background of broadly positive appraisal, students' cultural background was associated with differences in the relative strength of affective, cognitive, and arousal responses. Significant interaction effects indicated that Serbian and Bosniak students evaluated the same poems differently, not only in terms of valence and cognitive appraisal but also in arousal, with emotional and cognitive evaluations tending to align more strongly with the cultural origin of the selected narrative stimuli. Serbian students showed stronger arousal when engaging with the selected Marko Kraljević narrative, whereas Bosniak students showed a consistent preference for the selected Alija Đerzelez narrative in valence and cognitive evaluation without a corresponding increase in arousal. These findings suggest relative in-group enhancement within an overall positive evaluative context, rather than rejection of the other group's heroic figure. The two selected heroic figures, therefore, did not function as neutral literary figures in this sample, but as culturally situated symbols whose reception varied according to students' cultural background. While the study is limited by its sample size, regional focus, restricted stimulus set, and reliance on self-reported responses, its balanced mixed design and within-subject comparisons provide useful evidence for culturally patterned narrative engagement. These findings highlight the utility of affective and cognitive response measures for examining how specific identity-relevant narrative materials are processed in everyday educational settings. Future research should extend this approach to broader populations, additional heroic figures, multiple narrative excerpts, additional narrative forms, and multimethod assessments to further clarify how cultural storytelling continues to shape emotional and cognitive responses in educational and social contexts.

## Data Availability

The original contributions presented in the study are included in the article/supplementary material, further inquiries can be directed to the corresponding author.
